# Decidualized Stroma in Pelvic Lymph Nodes in a Pregnant Patient With Cervical Squamous Cell Carcinoma: A Case Report

**DOI:** 10.7759/cureus.11741

**Published:** 2020-11-28

**Authors:** Anoshia Afzal, Phillip Mingola, Michael Quinton, Roy Zhang

**Affiliations:** 1 Pathology, University of Oklahoma Health Sciences Center, Oklahoma City, USA

**Keywords:** decidualized, stroma, immunohistochemistry, pelvic lymph nodes

## Abstract

Decidualized endometrial stroma is an uncommon finding in lymph nodes but is typically found in the setting of endometriosis where endometrial glands give a hint toward the diagnosis. On the other hand, endometrial stroma with no identifiable endometrial glands can be challenging to differentiate from metastatic squamous cell carcinoma. We report a case of a 22-year-old female who presented to our medical center as a known case of cervical squamous cell carcinoma. The patient desired future fertility and became pregnant. She was treated during her second trimester and underwent a radical cesarean hysterectomy at 37 weeks’ gestation with bilateral pelvic lymph node dissection. Resection showed residual moderately differentiated squamous cell carcinoma of the cervix with lymphovascular invasion. Two pelvic lymph nodes were found to have decidualized stroma. Immunohistochemistry was done to rule out metastasis and no metastatic carcinoma was identified in any of the lymph nodes. It is necessary to be aware of the possibility of decidualized stromal changes in pelvic lymph nodes to avoid misdiagnosis.

## Introduction

Endometriosis is an extremely common finding in pelvic lymph nodes as a result of the deposition of endometrial glands and stroma in and around the pelvic organs. It is therefore not surprising to find decidualized stroma during pregnancy as a result of hormonal stimulation of this ectopic endometrial tissue [1]. However, decidualized stroma in the absence of endometrial glands can present a diagnostic challenge, especially during frozen sections to rule out metastases from endometrial or cervical carcinoma. Few cases have been reported in the literature associating decidualized changes with pregnancy in a patient with endometriosis and cervical carcinoma [1-3]. Our case is unique in that the patient has no history of endometriosis and never had signs or symptoms of endometriosis. We were able to identify decidual stromal changes and confirmed our findings with immunohistochemistry.

## Case presentation

**Figure 1:**
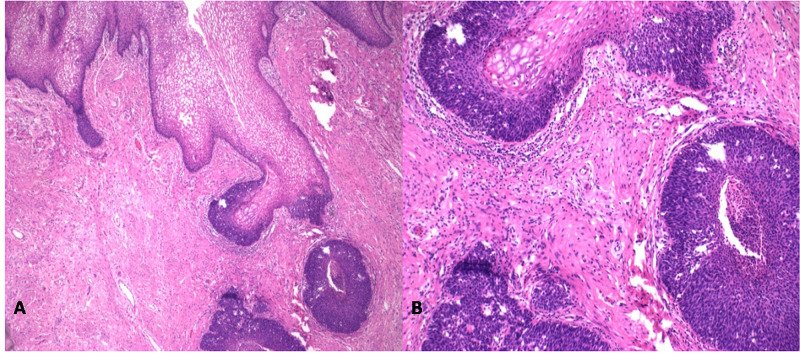
Hematoxylin and eosin staining (A: 4x and B: 10x) of sections of the cervix showed residual moderately differentiated squamous cell carcinoma.

We present the case of a 22-year-old woman without significant prior medical history who presented to our medical center after a loop electrical excision procedure (LEEP) cone biopsy at an outside hospital which showed cervical squamous cell carcinoma. The patient desired future fertility. A positron emission tomography (PET) scan did not show any evidence of distant disease. The patient became pregnant, was treated during her second trimester with cisplatin and paclitaxel, and underwent a radical cesarean hysterectomy at 37 weeks gestation with bilateral pelvic lymph node dissection. Intraoperative findings showed a clinically visible lesion on the cervix, but no visible lesions in the pelvis or lymph nodes. Resection showed residual moderately differentiated squamous cell carcinoma of the cervix, arising from severe dysplasia (Figure 1) with lymphovascular invasion. The bilateral parametria were negative for squamous cell carcinoma.

**Figure 2:**
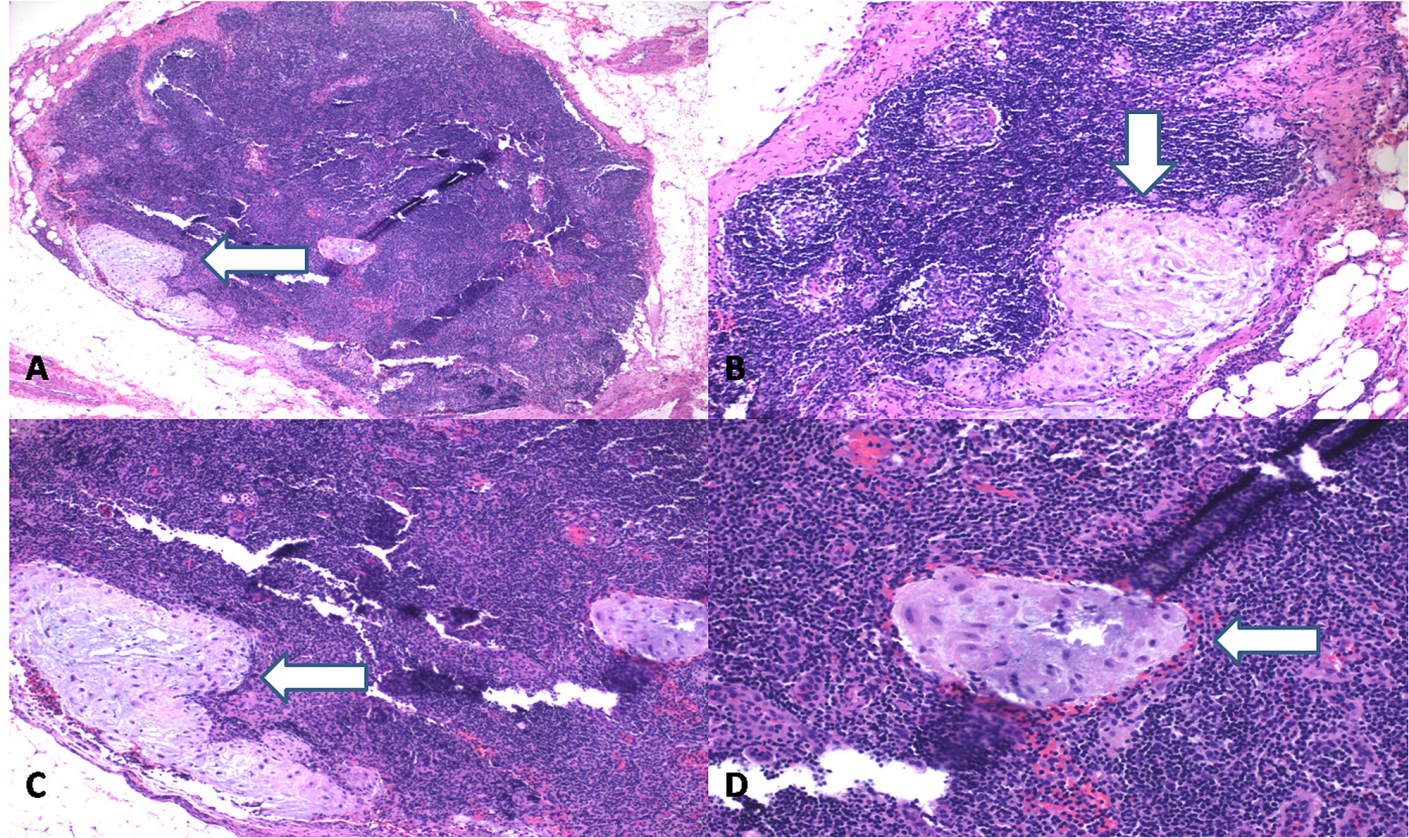
Hematoxylin and eosin staining (A: 4x, B-C: 10x and D: 20x) of pelvic lymph nodes showed clusters of large, polygonal cells with well-defined cell borders and abundant eosinophilic cytoplasm and round nuclei. No atypia was identified.

Fifteen pelvic lymph nodes were identified bilaterally. Two pelvic lymph nodes (one from each right and left side) had aggregates of large, polygonal cells with well-defined cell borders and abundant eosinophilic cytoplasm with round nuclei (Figure 2).

**Figure 3:**
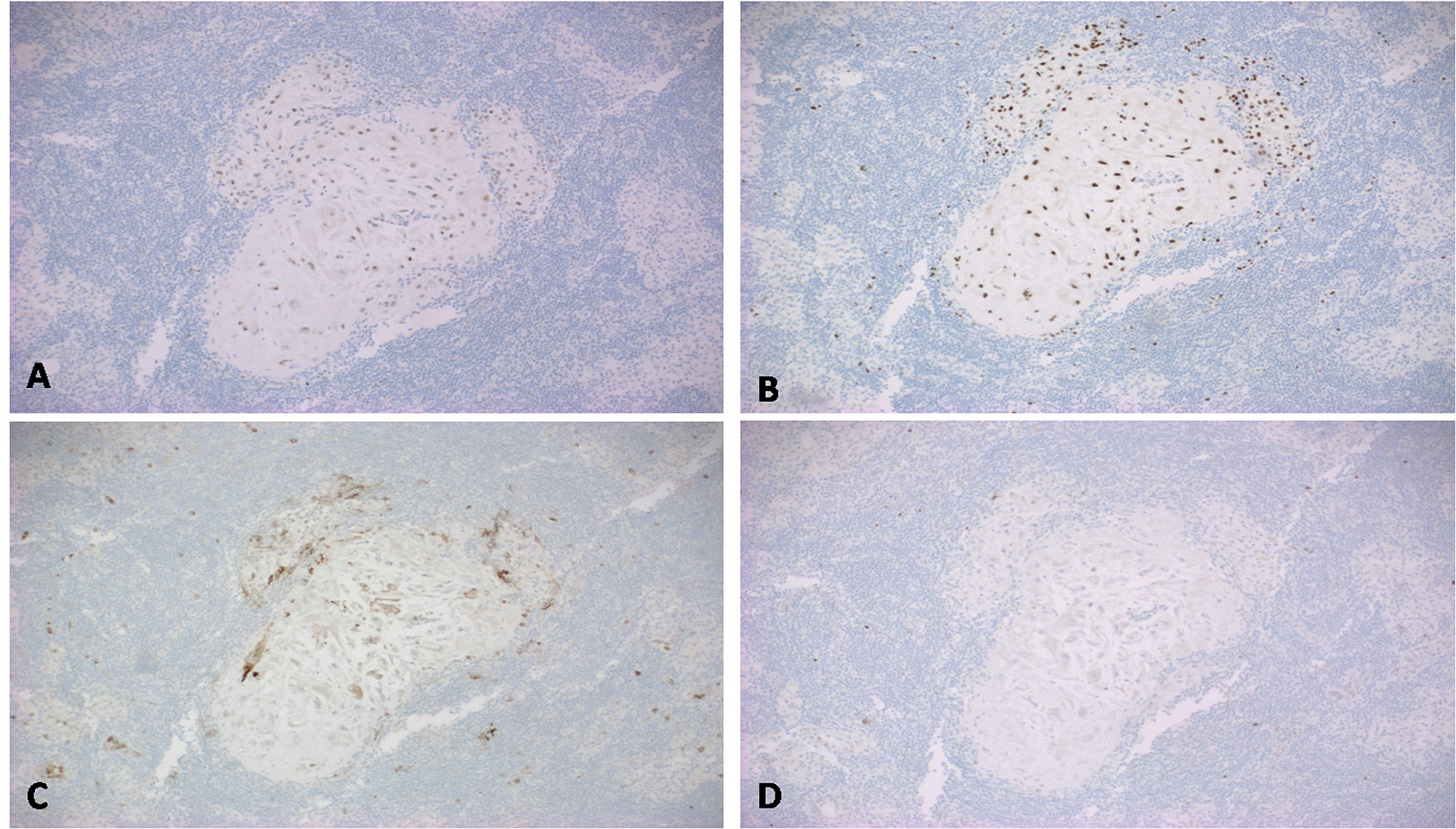
Immunohistochemical staining showed the cells to be positive for ER (Fig A), PR (Fig B) and CD10 (Fig C), thus confirming these clusters to be endometrial stromal cells. p63 (Fig D) was negative and ruled out metastatic squamous cell carcinoma. ER - estrogen receptor PR - progesterone receptor CD - cluster of differentiation p63 - transformation-related protein 63

These nests of loosely cohesive cells were positive for estrogen and progesterone receptors (Figure 3A, 3B), and a cluster of differentiation (CD)10 (Figure 3C) which is a sensitive and diagnostically useful marker for normal endometrial stroma. p63 (transformation-related protein 63) was negative (Figure 3D) and thus ruled out metastatic carcinoma.

The final diagnosis was endometrial stroma with decidual stromal changes in the lymph nodes. There was no evidence of involvement of the remaining specimen by endometriosis or ectopic decidual stroma.

## Discussion

This case presented a challenge in that although ectopic decidua does not have any clinical significance, it can become challenging in the setting of cervical and/or endometrial carcinoma where an intra-operative frozen section examination for staging purposes can lead to an incorrect diagnosis of metastatic carcinoma. The decidual stroma tends to be located in and around subcapsular sinuses and can mimic metastatic carcinoma. Several cases have been reported where this confusion led to a diagnostic challenge [1-3]. Decidualization in its simplest form is the conversion of normal endometrium into a specialized uterine lining under the influence of progesterone to accommodate gestation. The endometrial stroma becomes hypertrophied, and the endometrial lining becomes thickened. It is well known that decidual changes occur in pregnant patients with endometriosis as the ectopic glands also undergo similar changes as the endometrial lining [4,5]. This becomes problematic in situations when these ectopic glands appear as nodules and mimic carcinoma intraoperatively and it becomes necessary to rule out metastasis to avoid unnecessary and excessive interventions [6,7]. It is, therefore, important to be aware of the possibility of ectopic decidual changes and the fact that decidualized stroma will not have typical features of carcinoma, in particular, dysplasia, dyskeratosis, mitoses, and desmoplasia [8,9].

## Conclusions

Decidualized endometrial stroma presenting in a lymph node can mimic metastasis in the setting of squamous cell carcinoma, particularly when no glands are present to suggest endometriosis as a potential etiology. It is necessary to have a high index of suspicion of ectopic endometrial stroma, particularly in pregnant patients; immunohistochemical staining can be used to confirm the diagnosis.
